# Outcome and complication rate of total hip arthroplasty in patients younger than twenty years: which bearing surface should be used?

**DOI:** 10.1007/s00264-023-06086-0

**Published:** 2024-01-13

**Authors:** Sang Yoon Kang, Young-Seung Ko, Hong Seok Kim, Jeong Joon Yoo

**Affiliations:** Department of Orthopedic Surgery, Seoul National University Hospital, Seoul National University College of Medicine, Seoul, South Korea

**Keywords:** Total hip arthroplasty, Teenager, Complication, Survivorship, Bearing

## Abstract

**Purpose:**

Total hip arthroplasty (THA) in younger patients remains controversial due to concerns regarding long-term implant survival and potential complications. This study aimed to evaluate long-term clinical outcomes, complications, differences in complication and revision rates by bearing surfaces, and Kaplan–Meier survival curves for THA in patients under 20 years old.

**Methods:**

A retrospective review was conducted for 65 patients (78 hips) who underwent THA between 1991 and 2018. Their mean age was 18.9 years. Their clinical outcomes were assessed using the Harris Hip Score (HHS). Radiological outcomes were evaluated based on the presence of loosening, osteolysis, and heterotopic ossification. Complications such as dislocation, periprosthetic fractures, and infections were assessed. The mean follow-up period was 13.2 years (range, 5.0–31.2 years).

**Results:**

The mean HHS improved from 44.6 to 90.1. There were two cases of dislocation. However, no periprosthetic fracture, deep infection, or ceramic component fracture was noted. There were 19 revisions of implants. Eighteen of 19 hips were operated with hard-on-soft bearings in the index surgery (*p* < 0.01). The 23-year survivorship was 97.8% for THA using ceramic-on-ceramic bearings, while the 31-year survivorship was 36.7% using hard-on-soft bearings.

**Conclusion:**

THA in patients under 20 years old yielded promising clinical and radiological outcomes, although polyethylene-bearing-related concerns persisted. Previously operated patients with hard-on-soft bearing should be meticulously examined during the follow-up. As ceramic-on-ceramic bearing showed excellent survivorship in this particular cohort, we recommend the use of this articulation as the bearing of choice.

**Supplementary Information:**

The online version contains supplementary material available at 10.1007/s00264-023-06086-0.

## Introduction

Although clinical outcomes of total hip arthroplasty (THA) in teenagers have already been reported as favourable, there are many potential concerns related to the survival of implanted prostheses [1]. Hence, multiple surgical options have also been equally considered as viable options for young patients with debilitating hip diseases due to persistent concerns regarding the risk of early implant failure and limited longevity of THA [2]. However, hip resection arthroplasty and arthrodesis not only are functionally unsatisfactory, but also are deemed unacceptable choices for these patients with high functional demands [3, 4].

Thus, THA has been highlighted as the preferred surgical intervention. Lim et al. have reported excellent outcomes of THAs for 23 patients with secondary hip osteoarthritis resulting from Legg-Calvé-Perthes disease [5]. Favourable results of THA have also been demonstrated in various paediatric hip conditions, including sequelae of previous infections, juvenile rheumatoid arthritis, and slipped capital femoral epiphysis [6–8]. Especially, THAs offer young patients the opportunity to maintain a high activity level, endure repetitive loading, and regain their quality of life during this particularly important phase of life [9–13].

Historical data on cemented THAs have demonstrated an implant survival rate as low as 50% after 12–19 years [14, 15]. However, subsequent studies utilizing uncemented implants with ceramic-on-ceramic bearings have yielded more promising outcomes, showing a survival rate of 90% after ten years in patients under 20 years of age [16]. Nevertheless, there is a lack of comprehensive data on implant longevity and the choice of bearing surface in this particular cohort.

We hypothesized that all types of bearing surfaces could yield excellent functional and radiological outcomes in young patients undergoing THA. Therefore, the objectives of this study were to examine potential differences in complication and revision rates based on the bearing surfaces and to explore Kaplan–Meier survival curves within each cohort.

## Methods

### Patient demographics

**Fig. 1:**
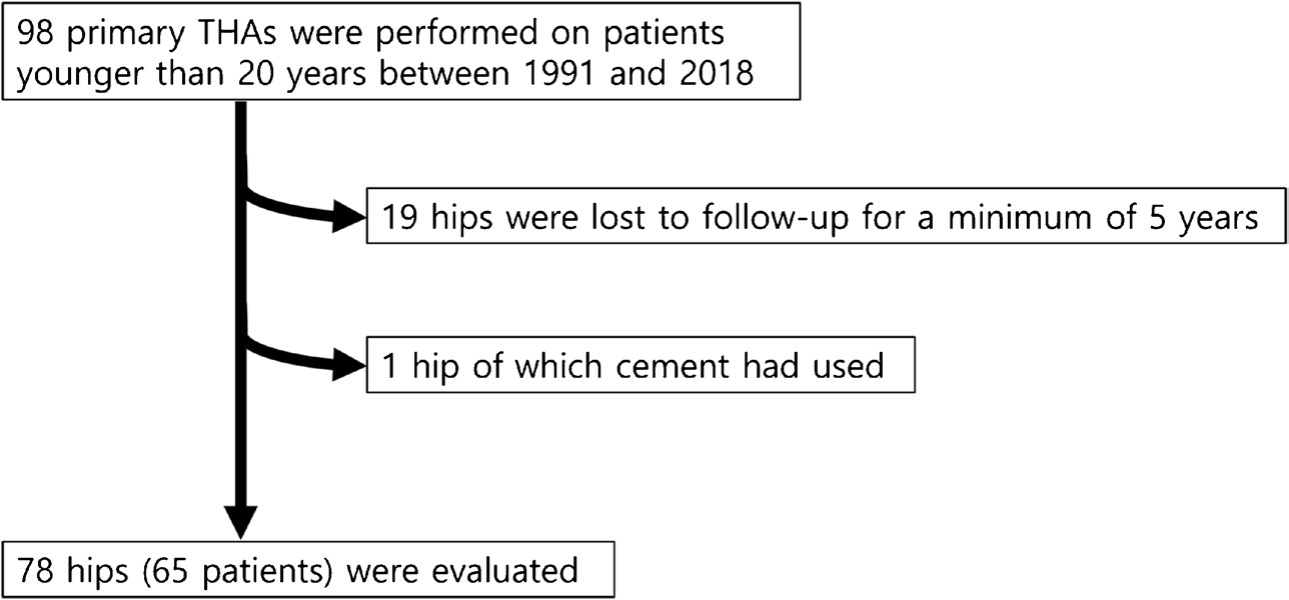
Flowchart showing the inclusion and exclusion of patients

This was a retrospective study. The study protocol was approved by the Institutional Review Board of our hospital (H-2305–111-1432). We included all patients who underwent a THA before the age of 20 years at the time of index surgery. From June 1991 to June 2018, we performed 98 THAs on the cohort whose age was younger than 20 years. Among them, 19 hips were lost to follow-up for a minimum of five years. One hip of which cement was used was excluded (Fig. 1). Thus, 78 hips were finally analysed. The final cohort included 36 (55.4%) females and 29 (44.6%) males aged between 13.3 and 20.0 years at the time of index operation. Their mean age was 18.9 years (Table 1).

**Table 1:** Demographics of the included patients

Patients (hips)	65 (78 hips)
Gender	
Male	29 (44.6%)
Female	36 (55.4%)
Age (years)	18.9 (range, 13.3–20.0)
Body mass index (kg/m^2^)	20.7 (range, 10.2–32.2)
Diagnosis (%)	
Osteonecrosis	28 (35.9%)
Sequelae of the previous infection	22 (28.2%)
Juvenile rheumatoid arthritis	10 (12.8%)
Sequelae of Perthes disease	7 (9.0%)
Multiple epiphyseal dysplasia	5 (6.4%)
Post-traumatic arthritis	3 (3.8%)
Sequelae of slipped capital femoral epiphysis	2 (2.6%)
Synovial chondromatosis	1 (1.3%)
Previous surgery (%)	
None	47 (60.3%)
In situ fixation	9 (11.5%)
Debridement	8 (10.3%)
Osteotomy	6 (7.7%)
Multiple drilling	6 (7.7%)
Arthrodesis	2 (2.6%)
Follow-up period (years)	13.2 (range, 5.0–31.2)

The most common underlying diagnoses were osteonecrosis of the femoral head in 28 (35.9%) hips and sequelae of the previous hip infection in 22 (28.2%) hips. All patients had failed conservative treatment which consisted of active and passive physiotherapy exercises and pain medications. Thirty-one hips underwent at least one operation before THA, which consisted of in situ fixation, osteotomy, and arthrodesis (Table 1).

### Surgical technique and implants

All index arthroplasties were performed at one institution by three high-volume hip arthroplasty surgeons (JJY and two surgeons who were not study authors). A posterolateral approach was used for 59 hips and a direct lateral approach was used for 19 hips with a lateral decubitus position. For acetabular components, Omnifit cup (Osteonics) in 25 hips, Bencox (Corentec) cup in 25 hips, Plasma cup (Aesculap) in 18 hips, Pinnacle cup (DePuy) in three hips, ABG cup (Howmedica) in two hips, Landos Atoll HA-coated acetabular cup (Landos) in two hips, Arthropor (Joint Medical) in one hip, Harris Galante II cup (Zimmer) in one hip, and Medinov hydroxyapatite-coated cup (Medinov) in one hip were used. All components were inserted in a press-fit manner. For femoral components, Secure-fit stem (Osteonics) in 25 hips, Bencox II (Corentec) in 21 hips, Bicontact (Aesculap) in 18 hips, Bencox M (Corentec) in four hips, Trilock (Depuy) in three hips, ABG stem (Howmedica) in two hips, Landos Euroform HA-coated (Landos) in two hips, S-rom (Joint Medical) in one hip, Multi-lock (Zimmer) in one hip, and Medinov stem (Medinov) in one hip were used. All polyethylene liners used in this study were ultrahigh molecular weight polyethylene (UHMWPE) sterilized with gamma irradiation in air.

### Postoperative care and follow-up visits

For the first six weeks after surgery, partial-weight-bearing with a crutch gait was recommended, followed by tolerable to full weight-bearing. Patients were followed-up for six weeks, six months, 12 months, and then annually after the surgery. No other specific physiotherapy was provided. The mean follow-up period was 13.2 years (range, 5.0 years to 31.2 years).

### Follow-up evaluations

Modified Harris hip score (HHS) was used to quantify initial functional impairment and measure improvement after surgery [17, 18]. Moreover, questionnaires and assessments were conducted to identify complications such as dislocation, ceramic-related noise, infection, and nerve injury.

Serial anteroposterior and lateral radiographs of the operated joint were reviewed by two independent observers (YSK and SYK) to assess the position of the prosthesis, loosening, calcifications, and osteolysis. Osteolysis was assessed using postoperative radiographs and CT images. Serial comparisons were meticulously conducted on postoperative radiographs. Osteolytic lesions were precisely localized based on the three DeLee and Charnley zones [19] on the acetabular side and the seven Gruen zones [20] on the femoral side. CT scans were performed in all instances where osteolysis was suspected [21]. Definitely loose components were defined as those that demonstrated a complete lucent line on any radiograph, a femoral subsidence of 2 mm or more, or an acetabular component migration or tilt [20, 22–25].

### Complication and revision rate based on the type of bearing surface in the index operation

The cohort was divided into two groups: a hard-on-soft bearing (ceramic- or metal-on-polyethylene bearing) group and a ceramic-on-ceramic bearing group. Basic demographic data, complication rate, and revision rate were compared between the two groups.

### Survivorship

We performed a Kaplan–Meier survival analysis with revision of any prosthetic component [26]. There was no reoperation without a change of prosthetic component.

### Statistical analysis

Continuous variables are presented as mean ± standard deviation. They were compared using Student’s t-test. Categorical data are presented as counts (percentages) and were analysed using chi-square or Fisher's exact test. *P*-value < 0.05 was considered statistically significant. Post hoc power analysis on the rate of reoperation of two groups was conducted. Statistical analyses were performed using SPSS Statistics for Windows version 25.0 (IBM Corp., Armonk, NY, USA).

## Results

### Cohort

In index surgeries, 29, 3, and 46 hips were operated with metal-on-polyethylene bearing, ceramic-on-polyethylene bearing, and ceramic-on-ceramic (CoC) bearing, respectively. There was no difference in age or BMI between the hard-on-soft bearing group and the CoC bearing group. Since CoC had been used in relatively recent periods, the follow-up period of the hard-on-soft bearing cohort was longer than that of the CoC cohort (Table 2).

**Table 2:** Analysis of demographics, complications, and clinical outcomes based on the bearing surfaces used in the index surgery

	Ceramic-on-ceramic bearing	Hard-on-soft bearing*	*p*-value
Number of hips (%)	46 (59.0%)	32 (41.0%)	
Age (years)	19.1 ± 1.4	18.7 ± 1.6	0.182
Body mass index (kg/m^2^)	20.8 ± 3.8	20.5 ± 3.6	0.715
Heterotopic ossification	3 (6.5%)	2 (6.3%)	0.669
Dislocation	0 (0%)	2 (6.3%)	0.165
Periprosthetic fractures	0 (0%)	0 (0%)	-
Ceramic component fractures	0 (0%)	0 (0%)	-
Periprosthetic joint infection	0 (0%)	0 (0%)	-
Polyethylene wear	-	16 (50.0%)	-
Periprosthetic osteolysis	0 (0.0%)	18 (56.3%)	< 0.001
Aseptic loosening	1 (2.2%)	18 (56.3%)	< 0.001
Revision of implants	1 (2.2%)	18 (56.3%)	< 0.001
Follow-up duration (years)	11.6 ± 5.8	15.5 ± 8.3	0.004
Modified Harris hip score	92.3 ± 9.8	86.9 ± 8.2	< 0.001

^*^Hard-on-soft bearing: ceramic-on-polyethylene bearing and metal-on-polyethylene bearing

### Clinical outcomes

The mean modified Harris hip score improved from 44.6 (range, 33 to 57) points preoperatively to 90.1 (range, 71–100) points at the final follow-up.

### Complications and revision surgeries

There were two hip dislocations during the follow-up. One hip dislocated after a fall from standing at postoperative three months, while the other patient had delayed dislocation at postoperative 18 years. They were treated successfully with closed reduction and abduction bracing for two months. There was no further dislocation.

Heterotopic ossification was observed in five (6.4%) hips, all of which were operated with a direct lateral approach. According to Brooker classification [25], three hips were grade I and two hips were grade II. No patient had any complaints with a functional range of motion.

There were no periprosthetic femoral fractures, periprosthetic joint infections, ceramic component fractures, or ceramic-related noises during the follow-up.

Gross polyethylene wear observed as an eccentric position of the femoral prosthetic head was present in 16 hips. Periprosthetic acetabular and femoral osteolysis was observed in 18 hips. Subsequent loosening of the prosthesis was detected in 19 hips (Table 2).

Nineteen hips with definitive loosening cases underwent revision total hip arthroplasty. In cases where both components were loosened due to extensive osteolysis or compatible bearings were unavailable, a total component revision was performed. Otherwise, revision of only the loosened component – either femoral or acetabular component—was performed. Specifically, total component revision for 11 hips (Supplementary Fig. 1), revision of acetabular components for seven hips, and revision of femoral component only for one hip were performed, all of which were attributed to peri-implant osteolysis. Among hips that underwent reoperation, the predominant bearing type used in the index operations was metal-on-polyethylene (17 hips, 89.5%), with one hip having ceramic-on-polyethylene bearing and one hip having ceramic-on-ceramic bearing. On average, revision surgery was performed at 7.4 years (range, 2.0–15.4 years) after the index surgery.

### Complication and revision rates based on the type of bearing surface

There were no significant differences in complications (such as heterotopic ossification or dislocation) that were not bearing-specific between groups. However, periprosthetic osteolysis was found to be more prevalent in the hard-on-soft bearing cohort (56.3%) than in the ceramic-on-ceramic bearing cohort (Supplementary Fig. 2). Aseptic loosening and subsequent revision of the implant were performed more in the hard-on-soft bearing cohort with statistical significance (Table 2). There was no difference in implant selection between patients who underwent revision surgeries and patients without revision surgery within the hard-on-soft bearing cohort. Modified Harris hip score was higher in the ceramic-on-ceramic bearing cohort than in the hard-on-soft bearing cohort (92.3 vs. 86.9, *p* < 0.001). Post hoc power analysis on the rate of reoperation of two groups demonstrated that the number of patients analysed was sufficient with a power larger than 80%.

### Survival

**Fig. 2:**
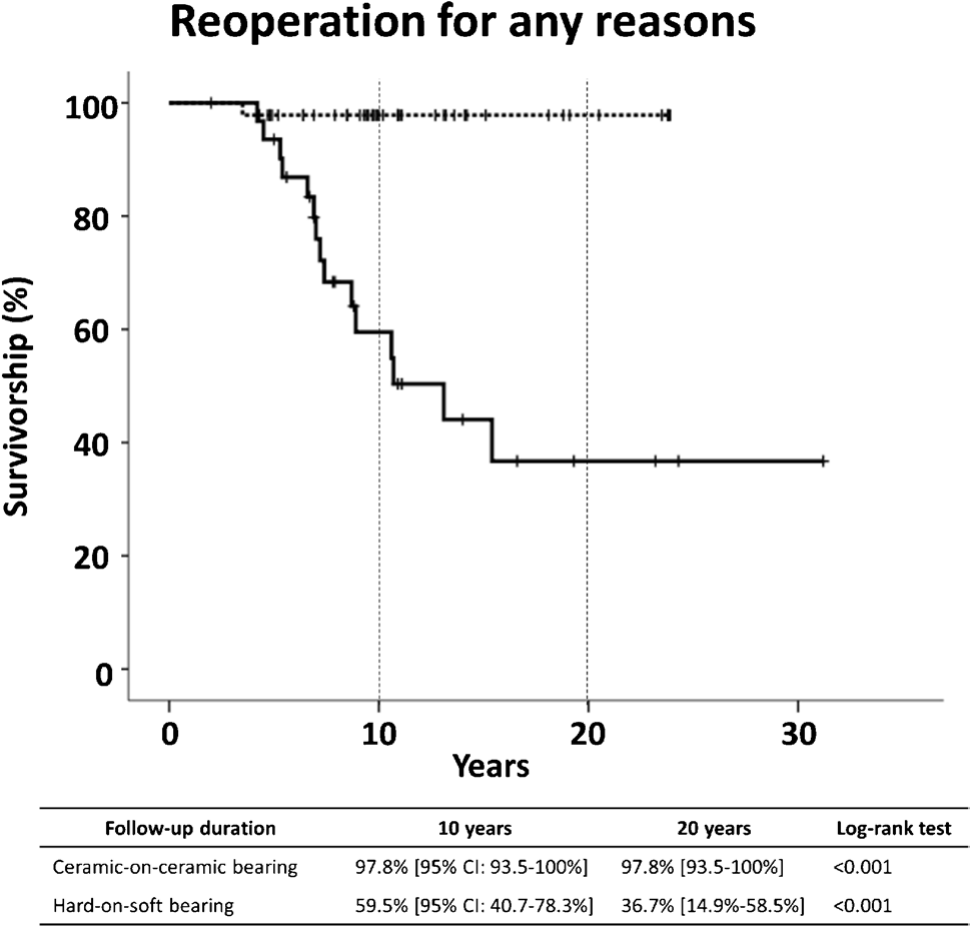
Kaplan–Meier curve with reoperation as the end point. Thick line indicates THA with hard-on-soft (metal-on-polyetheylene and ceramic-on-polyetheylene) bearing (*n* = 32), while dotted line indicates THA with ceramic-on-ceramic bearing (*n* = 46). Tick marks indicate censored data

Survivorship of THA using a ceramic-on-ceramic bearing with any reoperation as the endpoint was 97.8% (95% confidence interval [CI], 93.5% to 100%), while the survivorship of hard-on-soft bearing was 59.5% (95% CI, 40.7% to 78.3%) at postoperative ten years (*p* < 0.001, log-rank Mantel-Cox test). At postoperative 20 years, the survivorship of hard-on-soft bearing THA was 36.7% (95% CI, 14.9% to 58.5%), while that of ceramic-on-ceramic bearing THA was unchanged (*p* < 0.001) (Fig. 2).

## Discussion

THAs in young patients are demanding primarily attributed to their long life expectancy and expected high levels of sports. Concerns persisted regarding polyethylene wear, the presence of osteolysis, and aseptic loosening of THA using hard-on-soft bearings; in contrast, the use of ceramic-on-ceramic bearing THA showed promising results, as they reduced osteolysis with improved wear characteristics, leading to lower revision surgery rates.

Paediatric hip disease encompasses various disease entities. Previous studies reported that osteonecrosis and sequelae of childhood hip disease were predominant among patients who underwent THA [13, 27, 28], which was comparable to that of our study. The most common pathologies encountered in this study were osteonecrosis of the femoral head, followed by sequelae of previous infection, juvenile rheumatoid arthritis, and sequelae of Perthes disease.

Overall, our study demonstrated favourable results of THA in patients younger than 20 years. Similar to most other studies, we observed good to excellent improvements in functional outcome scores [10, 11, 28–33]. The modified Harris Hip Score was commonly used to assess clinical outcomes in these studies. Clohisy et al. have reported an increase in HHS from 42 to 83 points [33]. Finkbone et al. have documented a final HHS of 93.4 points [28]. Ozdemir et al. have recently demonstrated an increase in HHS from 51 to 81 points in young patients [12]. In our study, the mean modified HHS improved from 44.6 points (range, 33 to 57 points) preoperatively to 90.1 points (range, 71–100 points) at the final follow-up.

Due to young age of patients, complications and subsequent revision surgeries were inevitable. Although THA has become a reliable option for end-stage hip disease in recent times, it is rarely performed on adolescents mainly due to concerns about bearing wear, peri-implant osteolysis, and implant loosening. Additionally, reoperations related to infection, periprosthetic fractures, and implant failures must be taken into account. Clohisy et al. have reported a revision rate of 6.9% after an average follow-up of 4.2 years, utilizing various types of bearings [33]. Kamath et al. have reported a revision rate of 4.8% in 4.1 years of follow-up for 21 hips with various types of bearings [11]. In a study with a longer follow-up period, revision rates of 25.0% and 6.3% for acetabular component and femoral stem, respectively, were observed in 16 hips with 13.6 years of postoperative follow-up [10]. In the present study, the revision rate was higher than previously reported, with 19 (24.4%) out of 78 hips undergoing reoperation due to aseptic loosening of the implants.

The introduction of cross-linked polyethylene and advancements in sterilization methods have significantly enhanced the durability of polyethylene liners [34]. Highly cross-linked UHMWPE (HXLPE) was adopted for routine use in the early 2000s to reduce revision rates associated with wear, osteolysis, and aseptic loosening resulting from conventional UHMWPE wear. Since its inception, a substantial body of evidence consistently supports the utilization of HXLPE in THA, revealing notable reductions in wear rates and osteolysis. Paxton et al. have reported that at a 7-year follow-up, metal-on-conventional UHMWPE exhibits higher adjusted risks of all-cause revisions (hazard ratio [HR]: 1.75; 95% CI: 1.37–2.24; *p* < 0.001) and aseptic revisions (HR: 1.91; 95% CI: 1.46–2.50, *p* < 0.001) compared to metal-on-HXLPE [35]. A retrospective analysis by Hanna et al. focusing on patients aged 45–65 years with revision for polyethylene wear as the endpoint has shown an implant survivorship of 86% for conventional UHMWPE versus 100% for HXLPE at a minimum 13-year follow-up [36]. While CoC bearings exhibited superior performance compared to hard-on-soft bearings with a previous-generation polyethylene in the present study, further investigations are warranted to determine whether contemporary cross-linked polyethylene bearings would yield enhanced survivorship in this specific young patient population.

Moreover, a higher revision rate observed in this study might be attributed to excessive wear of traditional polyethylene in patients with higher activity levels. Concerns regarding elevated serum metal ion levels and wear of traditional polyethylene have prompted surgeons to explore alternative bearing options. Ceramic components have emerged as one such option, exhibiting good longevity in adult patients [37–40]. Notably, in young patients, ceramic-on-ceramic bearings have demonstrated outstanding outcomes, with Finkbone reporting only a 4.2% revision rate over 4.1 years of follow-up [28]. Trisolino et al. have reported a 2.7% revision rate [41] and Chapot et al. have reported no revision in 12 hips over an average follow-up of 6 postoperative years [13]. However, these results might be due to a relatively short follow-up period (Table 3). In our study, subgroup analysis revealed that patients operated with CoC bearings had a similar revision rate of 2.2%, with only one hip out of 46 requiring reoperation. Although ceramic component fracture remained a concern, no incidence of ceramic head or liner fracture occurred in our cohort.

**Table 3:** Literature review of total hip arthroplasty performed in patients under 20 years

Author	Journal – year	Number of patients (hips)	Data source	Average age (years; range)	Follow-up years (range)	Cement	Type of bearing surface (%)	Survival rate and outcomes	Complications	Revisions (%)
Bessette et al. [10]	Can J Surg. 2003	12 (16 hips)	Single center study	16.5 (10–20)	13.6 (10–25)	68.8% cementless 12.5% hybrids 12.5% cemented	MoP	HHS: 34.2 ➔ 97.2	2 D/L 1 IFF 1 NI	25.0% acetabular components 6.3% femoral stem
Restrepo et al. [30]	Acta Orthop. Belg. 2008	25 (35 hips)	Single center study	17.6 (13.5–20)	6.6 (4.2–10)	cementless	62.9% CoP 31.4% MoP 5.7% CoC	HHS: 51.9 ➔ 77.3 SF-36: PCS: 43.5 ➔ 63.8 SF-36: MCS: 58.5➔ 80.2	No complications	2.9% Bilateral polyethylene exchange
Clohisy et al. [33]	Clin Orthop Relat Res 2010	88 (102 hips)	Multi-center study	20 (12–25)	4.2 (2–16)	100% cementless acetabular component 95.1% cementless femoral component	74% MoP 14% CoC 7% CoP 5% MoM	HHS: 42 ➔ 83	4 D/L 1 IFF 2 NI 1 VD 1 infection	6.9% revisions
Finkbone et al. [28]	Journal of Arthroplasty 2012	20 (24 hips)	Single center study	16.4 (12–20)	4.3 (2.1–10.3)	cementless	CoC	HHS: 47.7 ➔ 93.4 Survival rate: 96%	1 AL 1 instability 1 screw rupture 1 NI	4.2% revisions
Kamath et al. [11]	Journal of Arthroplasty 2012	18 (21 hips)	Single center study	18 (13–20)	4.1 (2.1–7.4)	cementless	66.7% CoC 28.6% MoP 4.8% MoM	HHS: 43.6 ➔ 83.6	1 CCF	4.8% revisions
D'Ambrosi et al. [32]	Journal Orthop Sci 2016	24 (30 hips)	Single center study	19.7 (?-20)	12.5 (10–17)	cementless	60% CoC 40% CoP	HHS: 36.9 ➔ 92.3 WOMAC: 84.7 ➔ 28.5	1 IFF 4 HO	None
Tsukanaka et al. [31]	Acta Orthop. 2016	111 (132 hips)	National registry data	17 (11–19)	14 (3–26)	89% cementless acetabular component 95% cementless femoral component	98% CoP or MoP	Last HHS 83 10-year survival rate: 70%	19% acetabular osteolysis 21% femoral osteolysis	35.1% patients had at least 1 revision 14.4% patients had 2 or more revisions
Hannouche et al. [16]	Clin Orthop Relat Res 2016	91 (113 hips)	Single center study	17.3 (13.2–20.0)	8.8 (2–34.4)	92.0% cementless acetabular component 72.6% Cementless femoral component	92.9% CoC 7.1% CoP	HOOS score 79.3 ± 13.8 SF-121: PCS 48.1 ± 7.9 SF-121: MCS 47.4 ± 12.2 10-year survival rate: 90.3%	16 AL 1 SL	15.0% revisions
Halvorsen et al. [42]	Acta Orthop. 2019	747 (881 hips)	Multi-national registry data	18 (9–21)	N/A	74% cementless	32% MoP 21% CoP 17% MoM 11% CoC	10-year survival rate: 86% 15-year survival rate: 73%	61 AL 11 D/L 6 Infection	13.4% revisions
Trisolino et al. [41]	Children 2021	68 (74 hips)	Single center study	15.7 (11–18)	6.6 (2–20)	Cementless	CoC	HOOS score: 85 5-year survival rate: 97.6% 15-year survival rate: 94.4%	2 IFF	2.7% revision
Özdemir et al. [12]	Acta Orthop. 2023	96 (119 hips)	Single center study	20 (12–24)	11 (0–32)	Cemented	MoP	OHS: 24 ➔ 41 HHS: 51 ➔ 81 10-year survival rate: 99% 15-year survival rate: 88%	7 AL 3 D/L 3 SL	13.4% revisions
Chapot et al. [13]	Arthroplast Today 2023	11 (12 hips)	Single center study	16 (13–19)	6 (2–9)	Cementless	CoC	HHS: 81 OHS: 39.5		None
Present study		65 (78 hips)	Single center study	18.9 (13.3–20)	13.2 (5–31)	Cementless	59% CoC 37% MoP 4% CoP	HHS: 44.6 ➔ 90.1 23-year survival rate of CoC: 97.8% 31-year survival rate of CoP and MoP: 36.7%	2 D/L	53.1% revisions in CoP or MoP bearing 2.2% revisions in CoC bearing

*D/L* Dislocation; *IFF* Intraoperative femoral fracture; *NI* Nerve injury; *VD* Vessel damage; *AL* Aseptic loosening; *SL* Septic loosening; *CCF* Ceramic component fracture; *HO* Heterotopic ossification; *CoP* Ceramic-on-polyethylene; *CoC* Ceramic-on-ceramic; *MoP* Metal-on-polyethylene

Among revised hips, 18 out of 19 had initially been operated with hard-on-soft bearings. While there was no difference in the occurrence of dislocation or heterotopic ossification between the two groups, the incidence of wear-related complications was higher in the hard-on-soft bearing group. Notably, the revision rate in this cohort was comparatively higher, even when compared to other studies. This might be partly due to the longer follow-up period in our study, with an average of 7.4 years between the index surgery and the revision surgery, while most other previous studies on young patients had shorter average follow-up periods, typically less than seven years [11, 28–30, 41].

The survivorship of THA in patients under 20 years of age varied across studies. For instance, Tsukanaka et al. reported a ten year survival rate of 70% for 132 hips with hard-on-soft bearings, most of which were cementless [31]. Halvorsen et al. reported similar long-term survival rates of 86% at ten years and 73% at 15 years using various bearing options [42]. More recently, Trisolino et al. published results from 74 hips that underwent cementless THA with CoC bearings, demonstrating excellent overall survivor rates (97.6% at 5 years and 94.4% at 15 years) [41]. Özdemir et al. reported results from an average six year follow-up of cemented THA with metal-on-polyethylene bearings, showing survivor rates of 99% and 88% at ten years and 15 years, respectively [12]. In our study, the survivor rate of THA in young patients using CoC bearings was 97.8% (95% confidence interval [CI]: 93.5% to 100%) at postoperative 23 years, which was comparable to those of CoC THAs in other studies. However, the survivor rate of metal-on-polyethylene or ceramic-on-polyethylene bearings was 36.7% (95% CI: 14.9% to 58.5%) at 31 years. Such lower rates might be attributed to the extended follow-up and the use of traditional polyethylene.

The rate and subsequent survivorship of THA in patients with pediatric hip diseases may vary based on pre-existing hip pathology. Tan et al. recently reported that the rate of THA in patients with Perthes disease was 32% for those with a history of previous operative intervention and 40% for those without such history (*p* = 0.458) [43]. Similarly, in patients with a history of slipped capital femoral epiphysis, it was estimated that 45% of patients would undergo THA, with an overall revision rate of 11.9% manifesting at a mean of postoperative 6.5 years [44]. The rate of THA in patients with a previous hip infection during childhood varied. However, the revision rate was reported to be 8% in a recent study [45]. As illustrated above, the rate of THA may vary in different pediatric orthopaedic conditions. Thus, adequate patient stratification should be performed. However, the small number of subjects within a single centre impeded subsequent analysis. Future studies with registry data are warranted to minimize the bias.

This study has several limitations. First, this was a retrospective study with a relatively small sample size comprising 65 patients (78 hips), which might have a selection bias. Thus, caution is needed when interpreting findings of this study. However, the extended follow-up duration (mean: 13.2 years, range: 5.0–3.12 years) offered insights into longitudinal outcomes of THA in individuals under 20 years. Second, various confounding variables such as different implants and sizes, surgical approaches, and multiple surgeons were not meticulously assessed. Third, this study was conducted in an East Asian country where individuals frequently engaged in squatting and sitting on the floor, which might have increased the frequency of polyethylene wear. We also lacked information on activity levels of our patients, which could have a substantial impact on implant wear and longevity.

## Conclusions

THA in patients younger than 20 years old demonstrated favourable outcomes and advancements in clinical performance. However, concerns about the potential for osteolysis and aseptic loosening in cases with hard-on-soft bearings persisted as polyethylene wear emerged as the main factor for reoperations. Careful and thorough postoperative examinations are recommended, particularly for patients with hard-on-soft bearings. We recommend ceramic-on-ceramic bearing as the preferred surface option when performing THA in this specific cohort.

### Supplementary Information

Below is the link to the electronic supplementary material.

Supplementary Figure 1. A 19-year-old adolescent girl who had total hip arthroplasty 7 years ago due to sequelae of a previous hip infection showed extensive osteolysis around her acetabular component. (A) She had pain in inguinal and trochanteric areas. She was unable to walk without a walking aid. (B) All components were revised. An AP radiograph was obtained immediate postoperatively. (C&D) Follow-up radiographs and CT scans taken at 22 years showed well-fixed components. (PNG 2766 kb)

High resolution image (TIF 5057 kb)

Supplementary Figure 2. (A) An AP radiograph of an 18-year-old girl with her hip fused with screws due to tuberculosis. (B) Immediate postoperative radiograph after THA with ceramic-on-ceramic bearing. (C) Hip radiograph at postoperative 10 years. (D) Radiograph at postoperative 24 years. There was no evidence of prosthetic loosening, wear, osteolysis, or ceramic fracture. (PNG 2528 kb)

High resolution image (TIF 4968 kb)

## Data Availability

The data that support the findings of this study are available from the corresponding author, upon reasonable request.
