# Supplementary figures and images for: Outcome and complication rate of total hip arthroplasty in patients younger than twenty years: which bearing surface should be used?
Source: Int Orthop. 2024 Jan 13;48(6):1381–90. doi: 10.1007/s00264-023-06086-0 (PMC11076316; doi:10.1007/s00264-023-06086-0)

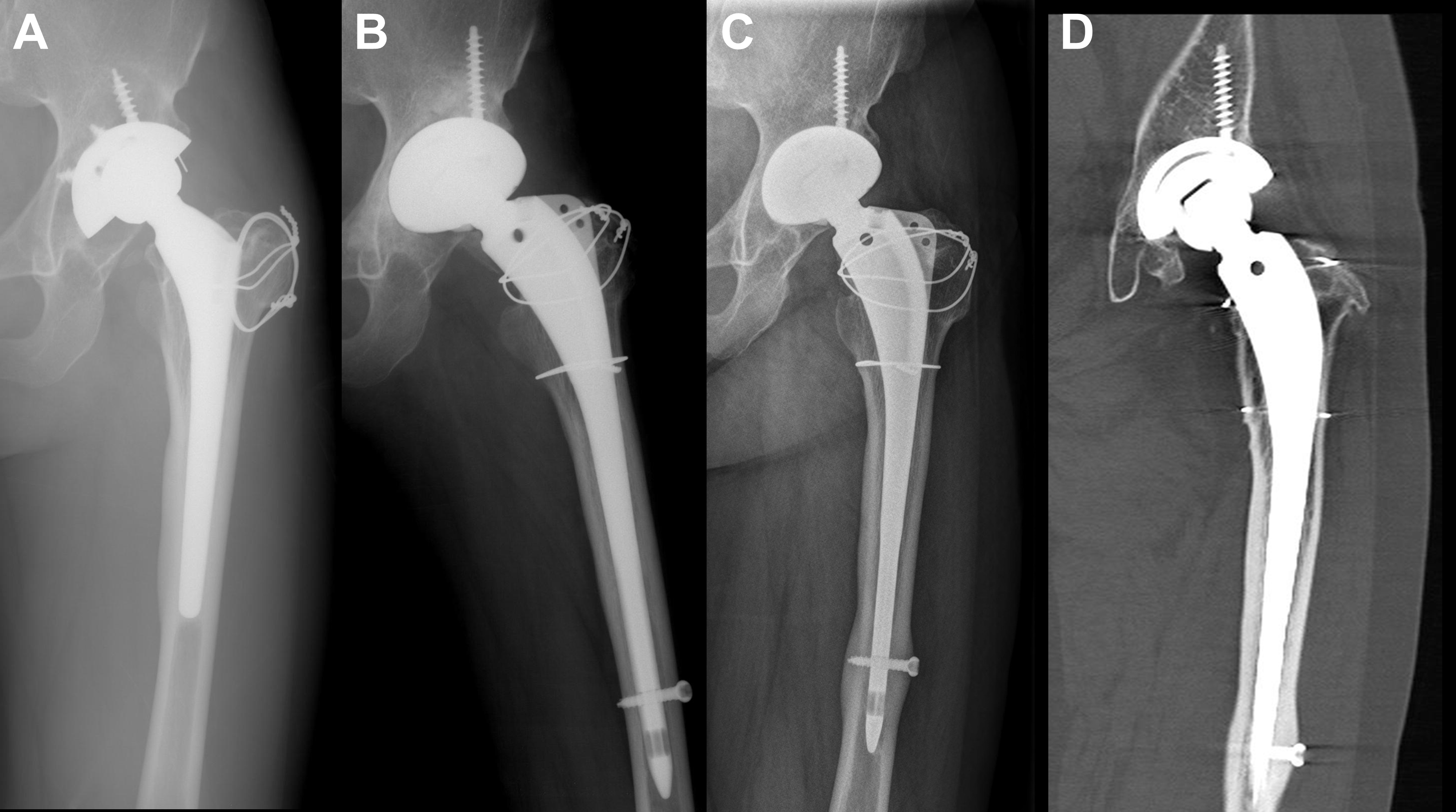

Supplement: Supplementary file 1 — Supplementary Figure 1. A 19-year-old adolescent girl who had total hip arthroplasty 7 years ago due to sequelae of a previous hip infection showed extensive osteolysis around her acetabular component. (A) She had pain in inguinal and trochanteric areas. She was unable to walk without a walking aid. (B) All components were revised. An AP radiograph was obtained immediate postoperatively. (C&D) Follow-up radiographs and CT scans taken at 22 years showed well-fixed components. (PNG 2766 kb) [file 264_2023_6086_Fig3_ESM.png]

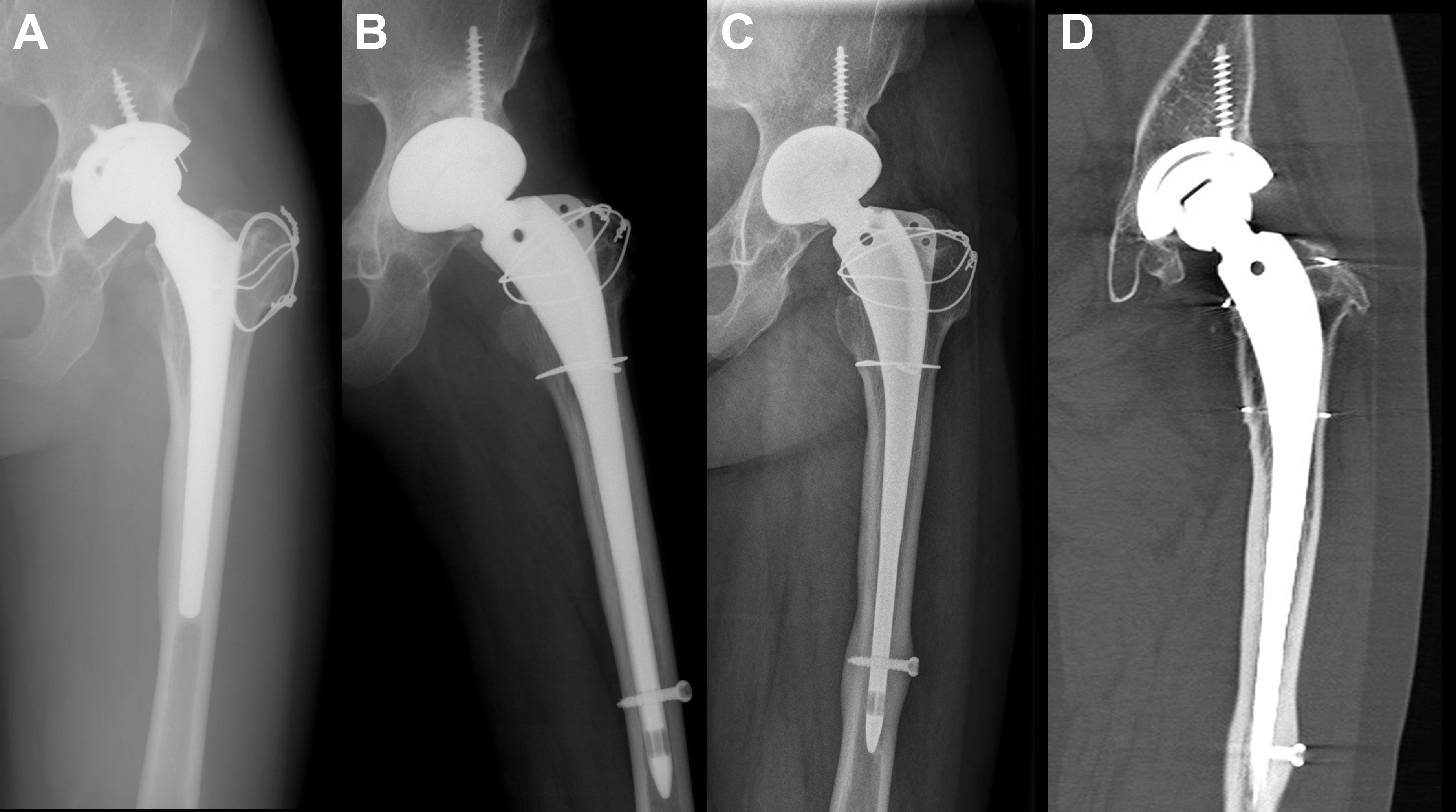

Supplement: Supplementary file 2 — High resolution image (TIF 5057 kb) [file 264_2023_6086_MOESM1_ESM.tif]

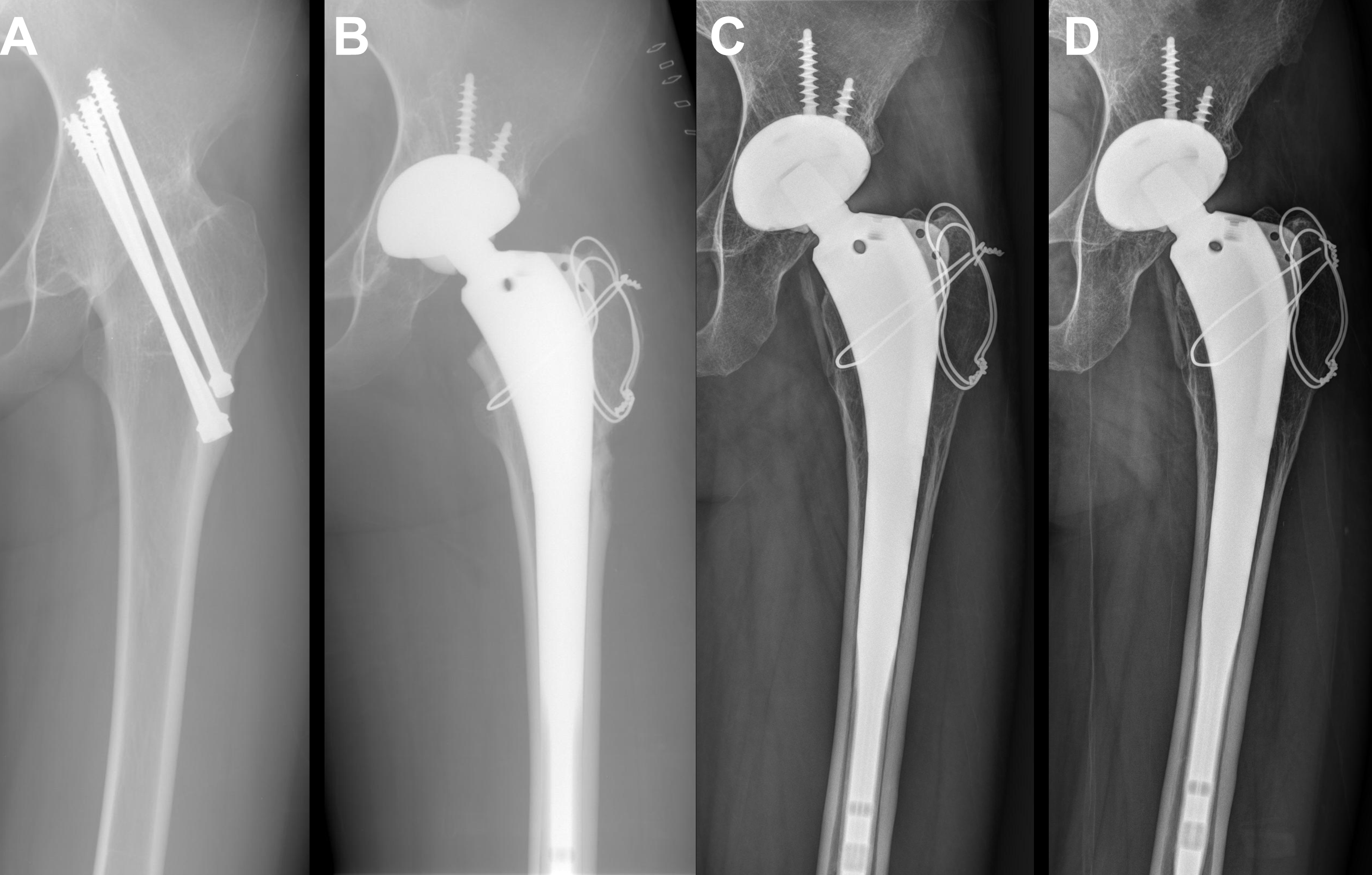

Supplement: Supplementary file 3 — Supplementary Figure 2. (A) An AP radiograph of an 18-year-old girl with her hip fused with screws due to tuberculosis. (B) Immediate postoperative radiograph after THA with ceramic-on-ceramic bearing. (C) Hip radiograph at postoperative 10 years. (D) Radiograph at postoperative 24 years. There was no evidence of prosthetic loosening, wear, osteolysis, or ceramic fracture. (PNG 2528 kb) [file 264_2023_6086_Fig4_ESM.png]

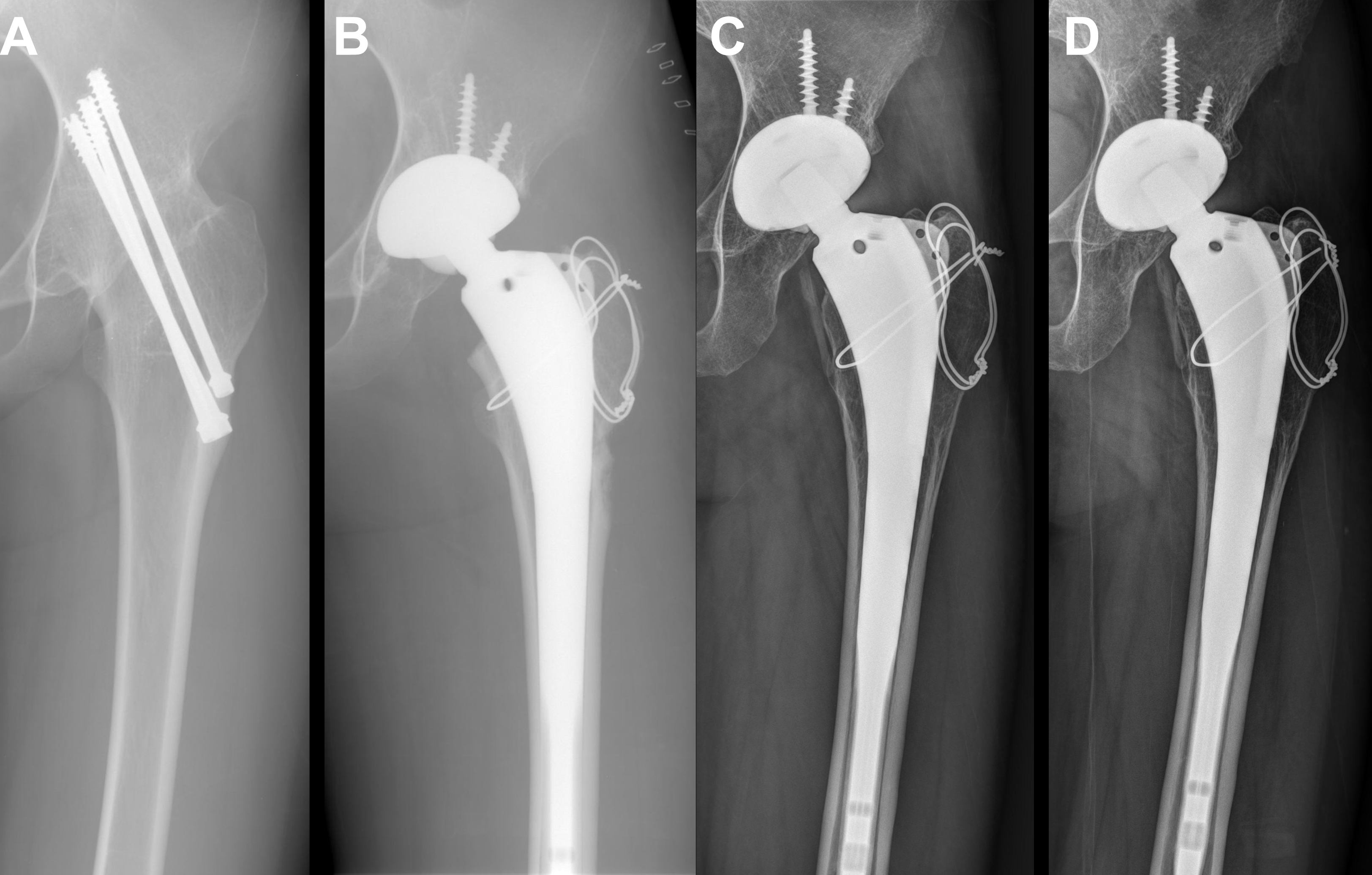

Supplement: Supplementary file 4 — High resolution image (TIF 4968 kb) [file 264_2023_6086_MOESM2_ESM.tif]
